# Supplementary material for: Analysis of PPARγ Signaling Activity in Psoriasis
Source: Int J Mol Sci. 2021 Aug 10;22(16):8603. doi: 10.3390/ijms22168603 (PMC8395241; doi:10.3390/ijms22168603)
Supplement: Supplementary file 1 [file ijms-22-08603-s001.zip › Supplemental materials_Analysis of PPARg signaling activity in psoriasis/Pathway models/Models images and html files/Model of PPARG related pathways in psoriasis (short version)/15633.html]

TICAM1


# Protein TICAM1

|  |  |
| --- | --- |
| URN | urn:agi-llid:10532 |
| Total Entities | 0 |
| Connectivity | 1168 |
| Name | TICAM1 |
| Description | toll-like receptor adaptor molecule 1 |
| Notes | This gene encodes an adaptor protein containing a Toll/interleukin-1 receptor (TIR) homology domain, which is an intracellular signaling domain that mediates protein-protein interactions between the Toll-like receptors (TLRs) and signal-transduction components. This protein is involved in native immunity against invading pathogens. It specifically interacts with toll-like receptor 3, but not with other TLRs, and this association mediates dsRNA induction of interferon-beta through activation of nuclear factor kappa-B, during an antiviral immune response. [provided by RefSeq, Jan 2012] |
| Primary Cell Localization | Cytoplasm |
| ObjectType | Protein |

---

|  |  |
| --- | --- |
| Pathway | Cortisol in Resolving Inflammation |
|  | NOD-like Receptors |
|  | Antiviral Signaling through Pattern Recognition Receptors |
|  | TLR4 Signaling in Leukocytes |
|  | T-Cell Independent B-Cell Activation |
|  | Toll-like Receptors in Sterile Inflammation |
|  | Necrosis |
|  | MC1R in anti-Inflammatory Signaling |
|  | Macrophage M1 Lineage |
|  | CD16/CD14 Proinflammatory Monocyte Activation |
|  | AXL Receptor Inhibits Macrophages and Dendritic Cells Function |
|  | Dendritic Cells Function in Atherosclerosis |
|  | Proteins Involved in Dilated Cardiomyopathy |
|  | Myocardial Infarction |
|  | MAVS in Antiviral Innate Immune Response in Myocarditis |
|  | Proteins Involved in Myocarditis |
|  | Toll-like Receptors (TLR) in Antiviral Innate Immune Response |
|  | Apoptotic Keratinocytes Clearance Recession in Systemic Lupus Erythematosus |
|  | Dendritic Cell Activation in Systemic Lupus Erythematosis |
|  | Plasmacytoid Dendritic Cell in Diabetes Mellitus Type 1 |
|  | TLR2 Signaling in Treg-Cell in Type 1 Diabetes (Animal Model) |
|  | Toll-like Receptors in beta-Cell |
|  | Celiac Disease |
|  | Crohn's Disease |
|  | Dendritic Cell Dysfunction in Crohn's disease |
|  | Dendritic Cell Function in Ulcerative Colitis |
|  | Nicotine anti-Inflammatory Effect in Ulcerative Colitis |
|  | Proteins Involved in Antiphospholipid Syndrome |
|  | TSLP Signaling in Bronchial Epithelial Cell in Asthma |
|  | Club-Cell Role in Asthma |
|  | Antigen-Presenting Cell Role in Asthma |
|  | Mucin Production in Goblet Airway Epithelial Cells |
|  | Airway Epithelial Cell Dysfunction |
|  | Monocytes Function in Gout |
|  | TLR2 Induced Synovial Fibroblast Activation in Rheumatoid Arthritis |
|  | Toll-like Receptors (TLR) in Periodontitis |
|  | IL1 Receptor Antagonist (IL1RN) Deficiency (DIRA) |
|  | PAPA Syndrome |
|  | TNF Receptor Associated Periodic Syndrome |
|  | Mevalonate Kinase Deficiency |
|  | Blau Syndrome |
|  | Cryopyrin (NLPR3) Associated Periodic Syndromes |
|  | Familial Cold Autoinflammatory Syndrome 2 |
|  | Familial Mediterranean Fever |
|  | Majeed Syndrome |
|  | Gluten Impact on Neuronal System (Hypothesis) |
|  | HMGB1 and IL1B in Neuroinflammation |
|  | Cholesteatoma |
|  | HPV E6 and E7 Induced Disruption of TNFR/NF-kB Signaling in Keratinocytes |
|  | TLR3 Role in Retinal Pigment Epithelial Cell |
|  | Proteins Involved in Otitis Media |
|  | Alveolar Macrophages Dysfunction in COPD |
|  | Mucin Hyperproduction in Goblet and Mucous Cells |
|  | NFKB Canonical Signaling Activation in Cancer |
|  | NF-kB Canonical Signaling |
|  | TLR3 Expression Targets |
|  | TLR4 -> NF-kB/IRF Expression Targets |
|  | TLR4 -> IRF Signaling |
|  | TLR3 -> IRF Signaling |
|  | TLR3 -> NF-kB Signaling |
|  | pathway 4 |
|  | Model of PPARG signaling in psoriasis |
|  | Model of PPARG related pathways in psoriasis (short version) |
|  | dsRNA induces innate immunity and INFs in alveolar cells |
|  | Dendritic Cell Activation in COVID19 |
|  | 1\_Insufficient activation of immune response in the middle ear epithelium cells in otitis media |
|  | 3\_Role of alveolar macrophages in pulmonary tissue destruction in COPD |
|  | 1\_Th2 cell response in asthma |
|  | 1\_Defects in response to pathogens in the gut promotes inflammation in Crohn's disease |
|  | 2\_Intestine epithelial cells drive immunological responses in ulcerative colitis |
|  | 1\_Intestine epithelial cell dysfunction in ulcerative colitis |
|  | 1\_Antigen-presenting cells promote islet dysfunction and immune system activation in T1D |
|  | 2\_Neuron and Schwann cell death |
|  | Model of PPARG signaling in psoriais (tested) |

---

|  |  |
| --- | --- |
| MedScan ID | 10532 |

---

|  |  |
| --- | --- |
| LocusLink ID | 10532 |
|  | 148022 |
|  | 106759 |
|  | 363328 |

---

|  |  |
| --- | --- |
| Alias | TICAM I |
|  | OTTMUSP00000017974 |
|  | TIR domain-containing adaptor protein inducing IFN-beta |
|  | toll interleukin 1 receptor domain containing adaptor inducing interferon beta |
|  | LOC148021 |
|  | LOC224899 |
|  | AW547018 |
|  | PRVTIRB |
|  | IIAE6 |
|  | TICAM1 |
|  | PRVTIRBs |
|  | CL24751 |
|  | proline-rich, vinculin and TIR domain-containing protein B |
|  | AW046014 |
|  | toll-like receptor adaptor molecule 1 |
|  | LOC193797 |
|  | TRIF |
|  | toll/interleukin-1 receptor domain-containing adaptor protein 1 |
|  | LOC363328 |
|  | TIR domain-containing adaptor molecule 1 |
|  | MGC35334 |
|  | TIR domain containing adaptor inducing interferon-beta |
|  | MyD88-3 |
|  | TICAM-1 |
|  | TIR domain-containing adapter protein inducing IFN-beta |
|  | putative NF-kappa-B-activating protein 502H |
|  | toll-interleukin-1 receptor domain-containing adapter protein inducing interferon beta |
|  | TIR domain-containing adapter molecule 1 |
|  | TIR domain-containing adaptor inducing interferon-beta |
|  | toll-interleukin 1 receptor domain-containing adapter protein inducing interferon-beta |
|  | TIR domain containing adapter inducing interferon-beta |
|  | toll-like receptor adapter molecule 1 |
|  | toll/interleukin-1 receptor domain-containing adapter protein 1 |

---

|  |  |
| --- | --- |
| GO ID | 0019901 |
|  | 0007249 |
|  | 0002756 |
|  | 0035666 |
|  | 0097190 |
|  | 0071222 |
|  | 0140052 |
|  | 0051607 |
|  | 0006954 |
|  | 0045087 |
|  | 0031663 |
|  | 0002281 |
|  | 0070266 |
|  | 0034128 |
|  | 0030890 |
|  | 0043123 |
|  | 0051092 |
|  | 0010508 |
|  | 0045080 |
|  | 1900017 |
|  | 0010628 |
|  | 0045359 |
|  | 0032755 |
|  | 0002735 |
|  | 0032816 |
|  | 0045429 |
|  | 0032092 |
|  | 0031398 |
|  | 0032760 |
|  | 0043496 |
|  | 0043330 |
|  | 0034138 |
|  | 0016032 |
|  | 0005776 |
|  | 0005829 |
|  | 0010008 |
|  | 0005739 |
|  | 0097342 |
|  | 0006915 |
|  | 0002376 |
|  | 0050871 |
|  | 0032481 |
|  | 0032496 |
|  | 0005737 |
|  | 0031410 |
|  | 0034142 |
|  | 0007165 |
|  | 0008624 |
|  | 0005515 |
|  | 0008063 |
|  | 0004871 |
|  | 0002224 |
|  | 0002755 |
|  | 0034130 |
|  | 0034134 |
|  | 0005886 |
|  | 0009615 |

---

|  |  |
| --- | --- |
| KEGG ID | hsa:148022 |
|  | mmu:106759 |

---

|  |  |
| --- | --- |
| Organism | Homo sapiens {Organism urn:agi-taxid:9606} |
|  | Mus musculus {Organism urn:agi-taxid:10090} |
|  | Rattus norvegicus {Organism urn:agi-taxid:10116} |
|  | Homo sapiens |
|  | Mus musculus |
|  | Rattus norvegicus |

---

|  |  |
| --- | --- |
| Mouse chromosome position | 17 |

---

|  |  |
| --- | --- |
| OMIM ID | 607601 |
|  | 614850 |

---

|  |  |
| --- | --- |
| Rat chromosome position | 9q12 |
|  | 9 |

---

|  |  |
| --- | --- |
| Hugo ID | 18348 |
|  | HGNC:18348 |

---

|  |  |
| --- | --- |
| Human chromosome position | 19p13.3 |

---

|  |  |
| --- | --- |
| Swiss-Prot Accession | Q8IUC6 |
|  | Q8IUC6.1 |
|  | Q80UF7 |
|  | Q80UF7.1 |
|  | B3Y691 |
|  | O75532 |
|  | Q86XP8 |
|  | Q96GA0 |
|  | Q3UDB7 |
|  | Q3UP66 |
|  | Q6P6J2 |
|  | Q8CIB7 |
|  | Q8JZV0 |

---

|  |  |
| --- | --- |
| GenBank ID | NC\_000019 |
|  | NM\_182919 |
|  | NP\_891549 |
|  | NG\_031998 |
|  | AC027319 |
|  | CQ871232 |
|  | CAH56797 |
|  | AB086380 |
|  | BAC55579 |
|  | AB093555 |
|  | BAC44839 |
|  | AB097023 |
|  | BAC77376 |
|  | AB446484 |
|  | BAG55261 |
|  | AF070530 |
|  | AAC28630 |
|  | AF492646 |
|  | AAO85488 |
|  | BC009860 |
|  | AAH09860 |
|  | BC035331 |
|  | BC136556 |
|  | AAI36557 |
|  | BC136557 |
|  | AAI36558 |
|  | BX349282 |
|  | CA308725 |
|  | DR000002 |
|  | Q8IUC6 |
|  | NC\_000083 |
|  | NM\_174989 |
|  | NP\_778154 |
|  | AC026385 |
|  | AB091053 |
|  | BAC55581 |
|  | AK054219 |
|  | AK083836 |
|  | AK086237 |
|  | AK089679 |
|  | AK143766 |
|  | BAE25531 |
|  | AK150150 |
|  | BAE29344 |
|  | AK155245 |
|  | BAE33144 |
|  | AK155906 |
|  | AK171578 |
|  | AK205957 |
|  | AK217234 |
|  | BC033406 |
|  | AAH33406 |
|  | BC037048 |
|  | AAH37048 |
|  | BC062191 |
|  | AAH62191 |
|  | BC094338 |
|  | AAH94338 |
|  | Q80UF7 |
|  | NC\_005108 |
|  | XM\_008766802 |
|  | XP\_008765024 |
|  | XM\_017596709 |
|  | XP\_017452198 |
|  | AC\_000077 |
|  | XM\_008757917 |
|  | XP\_008756139 |
|  | XM\_017603789 |
|  | XP\_017459278 |
|  | AABR07066529 |
|  | AAHX01058012 |
|  | NC\_018930 |
|  | ABBA01056040 |
|  | ABBA01056041 |
|  | AMYH02035304 |
|  | AMYH02035305 |
|  | AC\_000151 |
|  | NT\_011255 |
|  | NW\_927173 |
|  | NW\_001838477 |
|  | AC\_000062 |
|  | CR592986 |
|  | NT\_039649 |

---

|  |  |
| --- | --- |
| Swiss-Prot ID | TCAM1\_HUMAN |
|  | TCAM1\_MOUSE |
|  | B3Y691\_HUMAN |

---

|  |  |
| --- | --- |
| Cell Localization | Cytoplasmic vesicle |
|  | Autophagosome |
|  | Cytoplasm |
|  | Cytosol |
|  | Mitochondrion |
|  | Cytoplasmic vesicle, autophagosome |

---

|  |  |
| --- | --- |
| Ensembl ID | ENSG00000127666 |
|  | ENSP00000248244.4 |
|  | ENST00000248244.5 |
|  | ENSMUSG00000047123 |
|  | ENSMUSP00000055104.8 |
|  | ENSMUST00000058136.8 |
|  | ENST00000248244 |
|  | ENSP00000248244 |
|  | ENSMUSP00000055104 |
|  | ENSMUST00000058136 |

---

|  |  |
| --- | --- |
| MGI ID | MGI:2147032 |
|  | 2147032 |

---

|  |  |
| --- | --- |
| RGD ID | 1590868 |

---

|  |  |
| --- | --- |
| Unigene ID | Hs.29344 |
|  | Mm.203952 |

---

|  |  |
| --- | --- |
| Homologene ID | 8605 |

---

|  |  |
| --- | --- |
| IPI ID | IPI00170381 |
|  | IPI00216715 |
|  | IPI00398285 |

---
